# *Guzmania panamensis* (Bromeliaceae) – a new species from Talamanca Mountain in Veraguas province, Western Panama

**DOI:** 10.3897/phytokeys.25.5174

**Published:** 2013-07-25

**Authors:** Daniel A. Cáceres González

**Affiliations:** 1 Colaborator in the UCH Herbarium of the Universidad Autónoma de Chiriquí, David Chiriqui, Panama; 2 Herbarium Senckenbergianum Frankfurt/Main (FR), Germany. 0427 Estafeta Universitaria, David, Chiriquí, Panamá

**Keywords:** Epiphyte, Flora of Panama, Santa Fe National Park

## Abstract

*Guzmania panamensis* (Bromeliaceae), a new species so far endemic to Veraguas province, Western Panama, is described and illustrated. The new species is recognized due to its peduncle much longer than the leaves and its red floral bracts, shorter than the yellow flowers. The new species is compared to morphologically similar species, namely *Guzmania monostachia*, *Guzmania berteroniana*, *Guzmania elvallensis*, and *Guzmania skotakii*.

## Introduction

Bromeliaceae comprise 3,248 species and 58 genera (Luther 2010) which are grouped in subfamilies Brocchinioideae, Bromelioideae, Hechtioideae, Lindmanioideae, Navioideae, Pitcarnioideae, Puyoideae, and Tillandsioideae (Givnish et al. 2011).

According to Luther (2010), the genus *Guzmania* Ruiz & Pav. (Bromeliaceae) has more than 200 species and is one of the most diverse genera in Tillandsioideae.

In Panama, the Bromeliaceae comprise 206 species in 7 genera, of which *Guzmania* includes 45 species, 12 of them endemic (Cáceres González et al. 2011).

After the synopsis of the Bromeliaceae of Panama (see Cáceres González et al. 2011), it is clear that some areas of the country have not been sufficiently collected, principally in Western (Cordillera de Talamanca) and Eastern Panama.

During a botanical excursion made in December 2012, an interesting specimen of *Guzmania* was collected in a premontane rain forest from Santa Fe National Park (Cordillera de Talamanca) in the Veraguas province. These *Guzmania* plants are growing epiphytic, in the interior of the premontane rain forest, a few meters above the soil (close to the understory). It was found that the specimen did not match any of the known species of the genus and hence it is described and illustrated here as a new species of *Guzmania* from Western Panama.

## Taxonomic treatment

### Guzmania panamensis

Cáceres González sp. nov.

urn:lsid:ipni.org:names:77130424-1

http://species-id.net/wiki/Guzmania_panamensis

#### Diagnose.

This new species is similar to *Guzmania skotakii*, but it is characterized by shorter chartaceous leaves, arranged in an open rosette, with sheaths ovate-elliptic (9–11 × 3–5 cm) vs. longer leaves, coriaceous and erect, with sheaths broadly elliptic (20 × 10 cm). In *Guzmania panamensis* the floral bracts are obovate, acuminate 2.0–2.2 × 1.9–2.0 cm, shorter than the sepals, and petals are yellow; whereas in *Guzmania skotakii* the floral bracts are elliptic to oblanceolate, obtuse to subacute 3.0–4.0 × 1.6–2.5 cm, longer than the sepals, with cream colored petals.

#### Type.

**Panama.** Prov. Veraguas: Distr. Santa Fe, Santa Fe National Park, premontane rain forests, trail to Cascada Charco El Conejo close to the Quebrada Loma Grande and ANAM Station, 08°31'48.8"N, 81°08'58.8"W, at 767 m, 4 December 2012, D.A. Cáceres González *4385* (holotype: UCH; isotypes: FR and PMA).

#### Description.

**Figure 1.**
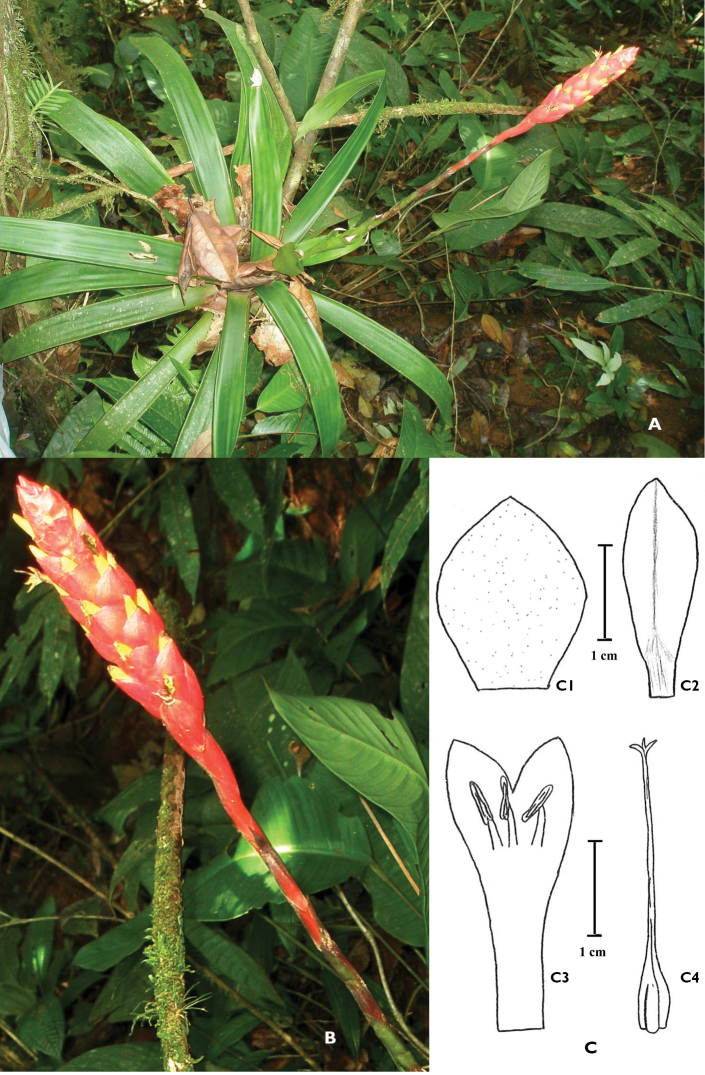
*Guzmania panamensis*. **A** Habit in Santa Fe National Park (province of Veraguas, Panama) **B** Inflorescence, bracts and flowers **C** Drawing of the flower parts: **C1** Floral bract, **C2** Sepal, **C3** Petals with filaments and anthers, **C4** Pistil. All from *D.A. Cáceres González 4385*. Photographs and drawing by Daniel A. Cáceres G.

Epiphytic herbs growing on the lower branches of the trees, close to the understory, flowering ca. 67 cm tall with the inflorescence extended. Leaves chartaceous, 40–55 cm long, forming an open rosette, green; leaf sheaths ovate-elliptic, 9–11 × 3–5 cm, with prominent longitudinal veins, with hyaline to membranaceous margins, lightly lepidote on the base with small brown scales in the inside and glabrous on the outside surface. Blades linear, ca. 30–45 × 2.8–3.8 cm (usually wider at the top half), strongly nerved in dry specimens (12–20 veins per cm), attenuate, long acuminate, green, glabrous. Peduncle elongate, erect, slender, 55 cm long, 5 mm in diameter, internodes (2.0–3.3 cm long), about equaling the leaves or slightly longer, hidden by the bracts, lepidote, dark brown. Peduncle bracts erect, much longer than the internodes, imbricate, chartaceous, 4.5–10.0 × 0.7–1.0 cm, bracts: the basal ones presenting a similar color to the leaves and glabrous, the distal ones reddish-green and lepidote, triangular ovate, acuminate to cuspidate. Inflorescence (excluding the peduncle) erect, 11.0 × 2.5 cm, simple, nearly cylindric, with about 25 flowers, polystichous, normally dense, all bracts bearing flowers. Rachis hidden, sparsely lepidote with minute lightly stellate brown to grayish scales. Floral bracts obovate, acuminate, erect, imbricate, globose, 2.0–2.2 × 1.9–2.0 cm (1 cm on the base), much longer than the internodes, shorter than the flowers, lightly nerved, lepidote at both surfaces, red. Flowers 3.6 cm long, sessile. Sepals joined for about one fourth (6–7/25 mm on the base) of their length, 2.5 cm long, 6–7 mm wide, yellow, asymmetric, elliptic, acuminate, coriaceous, erect, slightly fleshy on the base, with a strong central vein, glabrous. Corolla tubular, yellow, not fleshy, erect, 2.7–3.5 cm long (2.0–2.5 cm connate and adnate to the filaments), lobules 0.7–1.0 cm long and free, 0.4–0.6 cm wide, exceeding the sepals in 0.5–10 mm. Stamens alternate, and opposite to the lobule of the corolla, included, filaments all equaling in length, the free portion 3.5–4.0 mm long. Anthers dorsifixed at the middle. Pistil ca. 2.8–3.0 cm long, nearly as long as the petals, exceeding the stamens. Ovary ovate, 5 mm long, 2 mm in diameter, tapering from the base up to the style. Style slender, 2.5 cm long. Stigma trilobate, spreading, each lobe ca. 1.0–1.5 mm long. Capsule 2.5 cm long, 5 mm in diameter. (Fig. 1)

#### Distribution.

*Guzmania panamensis* is only known from Santa Fe National Park, Veraguas province in the Cordillera de Talamanca, Panama.

#### Ecology.

The new species grows in primary forest or old secondary forest, usually close to the understory and premontane rain forests at altitudes around 750 m. The common associated plant species are *Guzmania musaica* (Linden & André) Mez (Bromeliaceae), *Zamia* sp. (Zamiaceae), *Palicourea guianensis* Aubl. (Rubiaceae), *Danaea nodosa* (L.) Sm. (Marattiaceae), and *Dichaea* sp. (Orchidaceae). The type specimen of the new species has been collected as epiphyte on a height of one m above the ground on a shrub of *Psychotria* sp. (Rubiaceae) (Fig. 1A). It was observed flowering in December (start of the dry season).

#### Etymology.

*Guzmania panamensis* is named in honour of Panama country.

#### Conservation status.

*Guzmania panamensis* has been collected only once in Panama. In the type locality area, only three individuals were observed and hence it is assumed to be uncommon. No other specimen could be found among the material that has been collected for seventy years, as part of the project Flora of Panama of the Missouri Botanical Garden, supporting the view that this taxon is rare even inside this national park. Similar habitats, surrounding the park have been visited by the author and no specimen similar to *Guzmania panamensis* has been found in such areas. Therefore, in the conservation assessment presented here (following IUCN 2001 guidelines), *Guzmania panamensis* is categorized as Critically Endangered (CR), represented by only a single distribution record and based on the criterium B2biii.

#### Observations.

Four species have a close resemblance with *Guzmania panamensis*: *Guzmania monostachia* (L.) Rusby *ex* Mez, *Guzmania berteroniana* (Schult. & Schult. f.) Mez, *Guzmania elvallensis* Luther, and *Guzmania skotakii* Luther.

This new species resembles *Guzmania monostachia* (see Smith and Downs 1977, Mez 1896, Utley 1994), but differs from it by its larger size of the plant and the color of the floral bracts. *Guzmania panamensis* when flowering reaches about 67 cm tall vs. 20–40 cm in *Guzmania monostachia*. The peduncle of the new species is much longer than the leaves with red floral bracts and yellow flowers; while in *Guzmania monostachia* the peduncle is much shorter, the floral bracts are bright red or rarely white, and the flowers are usually white with conspicuous brown longitudinal stripes.

In *Guzmania berteroniana* the floral bracts exceed the sepals, and the sepals are 22 mm long (connate for 2 mm); while in *Guzmania panamensis* the floral bracts are shorter than the flowers (including the sepals), and the sepals are 25 mm long (connate for 6–7 mm).

This new species differs from the similar *Guzmania elvallensis* (Luther 1996), by the color of its petals (yellow vs. pale green). Another important feature is that in *Guzmania panamensis* the floral bracts are red, whereas in *Guzmania elvallensis* they are green.

*Guzmania panamensis* differs from the similar *Guzmania skotakii* (Luther 1991), by its smaller leaves, chartaceous in open rosette with sheaths ovate-elliptic (9–11 × 3–5 cm) vs. longer leaves, coriaceous and erect, with sheaths broadly elliptic (20 × 10 cm). In the first species the floral bracts are obovate, acuminate 2–2.2 × 1.9–2 cm, shorter than the sepals, with yellow petals; whereas in the second species the floral bracts are elliptic to oblanceolate, obtuse to subacute 3–4 × 1.6–2.5 cm, longer than the sepals, with cream petals.

## Supplementary Material

XML Treatment for Guzmania panamensis
